# Myofibre segmentation in H&E stained adult skeletal muscle images using coherence-enhancing diffusion filtering

**DOI:** 10.1186/1471-2342-14-38

**Published:** 2014-10-29

**Authors:** Harry Strange, Ian Scott, Reyer Zwiggelaar

**Affiliations:** Department of Computer Science, Aberystwyth University, Penglais Campus, SY23 3DB Aberystwyth, UK; Department of Cellular Pathology, Nottingham University Hospitals NHS Trust, Queen’s Medical Centre, NG7 2UH Nottingham, UK

**Keywords:** Digital pathology, Muscle biopsy, Image segmentation

## Abstract

**Background:**

The correct segmentation of myofibres in histological muscle biopsy images is a critical step in the automatic analysis process. Errors occurring as a result of incorrect segmentations have a compounding effect on latter morphometric analysis and as such it is vital that the fibres are correctly segmented. This paper presents a new automatic approach to myofibre segmentation in H&E stained adult skeletal muscle images that is based on Coherence-Enhancing Diffusion filtering.

**Methods:**

The procedure can be broadly divided into four steps: 1) pre-processing of the images to extract only the eosinophilic structures, 2) performing of Coherence-Enhancing Diffusion filtering to enhance the myofibre boundaries whilst smoothing the interior regions, 3) morphological filtering to connect unconnected boundary regions and remove noise, and 4) marker controlled watershed transform to split touching fibres.

**Results:**

The method has been tested on a set of adult cases with a total of 2,832 fibres. Evaluation was done in terms of segmentation accuracy and other clinical metrics.

**Conclusions:**

The results show that the proposed approach achieves a segmentation accuracy of 89% which is a significant improvement over existing methods.

## Background

The examination of stained biopsies from skeletal muscle is a vital component in the diagnostic pathway for the vast majority of neuromuscular disorders and remains essential as a preliminary investigation despite the availability of electron microscopic, genetic, and molecular tests for specific conditions. Detection of variation in fibre size is seen as a first step in the diagnosis of the majority of neuromuscular disorders including myopathic, dystrophic, neurogenic, and inflammatory conditions; examples of which include mitochondrial cytopathies, muscular dystrophies, motor neuron disease, and dermatomyositis [1, 2]. Muscle biopsies in which there is no fibre size variation are most often normal [3]. Further analysis of fibre size variation for those which exhibit a continuous distribution or biopsies showing a biphasic pattern can further assist in obtaining a diagnosis. When performing analysis on myofibre images it is useful to identify the myofibre boundary so that morphometric measures can be obtained. Manual identification and measurement of myofibres and their boundaries can be time consuming, error prone, and can often suffer from intra and inter operator variability. These drawbacks have led to the development of various techniques for automatic myofibre segmentation.

**Figure 1:**
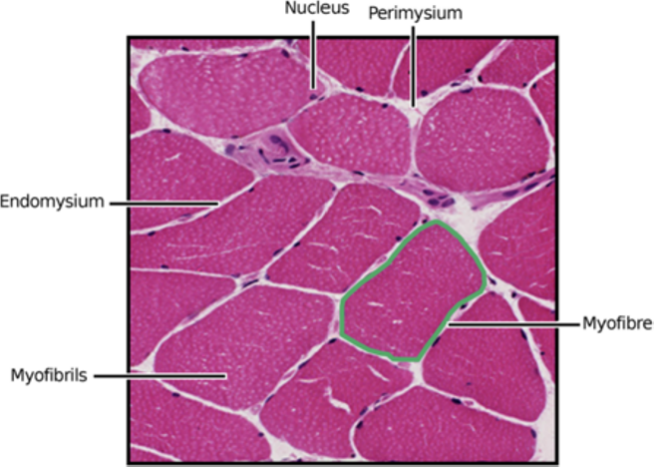
**General organisation of skeletal muscle.** Polygonal myocytes are shown revealing the presence of peripheral nuclei. The stained sarcoplasm within the myofibrils is light pink. Each fibre is surrounded by mesenchymal matrix known as the endomysium. Myocytes are grouped into fascicles invested by a connective tissue sheath known as perimysium. The perimysium contains nerves and blood vessels that supply the muscle.

One of the key steps for such automatic methods is the accurate segmentation of the myofibres from the surrounding connective structures (Figure 1). Inaccuracies in the segmentation of the myofibre boundary will lead to errors at later stages of analysis. As such, there is a need for automatic segmentation methods that provide accurate results and are robust to different imaging conditions. There are numerous existing approaches to automatic myofibre segmentation ranging from simple thresholding [4] to more advanced methods that use deformable models [5]. These approaches either identify the pixels in the image associated with the fibres themselves or identify the boundaries of the fibres — the perimysium and endomysium (see Figure 1). However, these methods may fail in cases where there are weak fibre boundaries or an increased presence of noise due to inconsistencies in the staining process.

The simplest automatic approach is to perform thresholding to segment the image into foreground and background regions, one corresponding to myofibres and the other representing background and connective tissue. The central step of such an approach is the selection of a threshold value to separate the two regions such that they are well represented. Although methods exist to estimate the threshold automatically [6], or manually [4], thresholding is an inefficient method for myofibre segmentation as the fibres cannot be split from the background, or from each other, using a single threshold value. As such, more advanced methods are needed to segment the myofibre regions.

These more advanced approaches can be broadly split into two categories: those that use edge detection to identify the perimysium or endomysium (the myofibre boundaries), and those that use deformable models to identify the myofibres. This dichotomy is by no means exclusive as many approaches will use methods from both of these categories.

Edge detection methods seek to segment the perimysium and endomysium so that the fibres can be segmented from these boundaries. Therefore, edge detection methods are often used as a pre-processing step to fibre segmentation with the justification being that, if the boundaries are correctly identified, the fibres themselves can be segmented as the interior of the boundary regions. Tzekis et al. [7] presented an ensemble approach to edge detection, with Sobel, Laplacian, and the Mode Colour algorithms being employed in a single framework to attempt to robustly identify the myofibre boundaries. Once identified, the edges could be used as *a priori* information for further segmentation; it is worth noting however that the approach to such a segmentation is not mentioned in the original paper. Sertel et al. [8] present a more established framework that uses edge detection as the first step in myofibre segmentation. Ridge analysis [9] is applied to ATPase stained cross-sections to segment the connective tissue so that thresholding the ridge likelihood image produces a rough segmentation of the connective tissue regions. This rough segmentation is further refined through morphological operations to connect any unconnected components. Small regions are removed and the watershed transform is used to separate any touching boundaries. The results show that using this approach myofibres can be effectively segmented with the added benefit of the algorithm being fast to execute.

The second category of segmentation method seeks to apply deformable models, such as active contours [10], to identify the myofibre regions. Active contour models require a seed point or curve to initialise the segmentation, as such, many of the active contour based methods can be distinguished by the heuristic used to identify the initial seed locations. The simplest approach is to use manual seed locations to identify the central points of the myofibres. Such an approach was presented by Klemenčič et al. [11], where the user manually identifies the central regions of each myofibre. A Voronoi polygon is then formed from these centroids and a modified active contour model is applied to refine the Voronoi polygons so that they ‘fit’ the myofibres. An attempt to automate that initialisation process was made by Röhrle et al. [5] who used the Hough transform to identify circular regions in the image. This method is however sensitive to noise and a parameterisation of the acceptable range of circular radii is needed. The limitations of standard active contour models was addressed by Kim et al. [12] where a level set approach was applied to improve the active contour segmentations. Level sets were used to extend the active contour framework by incorporating colour and texture information. As well as this, the initial contour is estimated using a heuristic thresholding approach to overcome the problem associated with manually identifying seed positions. A recent approach by Mula et al. [13] uses Gradient Vector Flow (GVF) to delineate myofibre boundaries. GVF seeks to improve upon active contour modelling by using the gradient vector flow of an image to pull the active contour towards the object boundary. The GVF based myofibre segmentation method initially uses ridge detection to enhance the boundaries before seed locations are automatically chosen by identifying concave areas. The GVF deformable model is then applied to segment the myofibre regions.

One of the main limitations of the above methods is that they can often fail in cases where noise is introduced as a result of the staining or sectioning process, and also in cases with weak boundaries. As well as this, there is no previous work that directly compares the different approaches to myofibre segmentation in adult cases. Motivated by these drawbacks, this paper presents a method for myofibre segmentation based on Coherence-Enhancing Diffusion (CED) filtering [14] that is robust to noise and weak fibre boundaries, and also provides a side by side comparison with the main approaches to the segmentation problem. CED has been successfully applied to numerous image analysis problems such as axon tracking [15], image in-painting [16], fringe pattern demonising [17], and lumen segmentation [18]. The basic principle of CED filtering is to improve the quality of the flow like structures in an image through the use of anisotropic diffusion tensors. Unlike existing line enhancement methods such as that of Frangi et al. [9], CED filtering uses the structure tensor, along with its principal eigenvectors, to define the orientations for smoothing. CED is therefore well suited to myofibre segmentation as it is robust to noise and can enhance weak fibre boundaries.

Therefore, the main contributions of this work are twofold. First, a new method for myofibre segmentation is presented using a multi-step process based upon Coherence-Enhancing Diffusion filtering [14]. This new method significantly out-performs existing approaches when segmenting H&E stained adult skeletal myofibres. Second, existing approaches to myofibre segmentation are compared and contrasted for the first time, thus giving an overview of the comparative performance of methods when seeking to segment myofibres in H&E stained adult cases.

## Methods

### Myofibre normal cases

The muscle tissue, obtained following open or Bergstrom needle biopsy, was frozen onto cork blocks and sectioned with the fibres orientated in the transverse plane (5 - 7 *μm*) using a frozen cryostat (Leica CW1900, Leica GmbH, Germany). The sections were then placed on glass slides and stained with haematoxylin and eosin (H&E) using standard protocols [3]. Entire sections, obtained from 10 anonymised normal adult patients, were scanned at 400 × magnification using a NanoZoomer Digital Pathology System (Hamamatsu Photonics UK) and the resulting images viewed using NDP Software (NDP.view 2, Hamamatsu Photonics UK). The final image data used for experimentation was taken as regions of interest measuring 1030×1300 pixels (926 × 1169 *μm*) viewed at 10× zoom and stored as uncompressed TIFF files. A total of 2832 fibres were identified across the region of interest images.

### Ground truth

The ground truth data was generated by a single user (HS) tracing the boundaries of the fibres across all images. In order to validate the manual segmentations as ground truth, a repeatability study was performed whereby the same image was segmented 10 times by the same person. This process resulted in multiple ground truth regions which were then co-registered using a group-wise registration algorithm [19] to obtain an aligned ‘mean image’. The absolute difference between each manual segmentation and the mean image was then computed to obtain an overall measure of the ground truth repeatability error. The error was measured as the number of pixels in the difference image divided by the total number of pixels in the original manual segmentation mask. The average error across all 10 cases was measured as 0.056 (±0.01). This error is low enough to accept the manual segmentations as a good representative of ground truth as it shows that the estimated error in the manual segmentations is only 0.056.

### Overview of method

This work uses Coherence-Enhancing Diffusion (CED) filtering to segment the boundary regions whose interiors contain the myofibres. CED filtering [14] seeks to smooth a degraded original image along the directions determined by the image’s structures through the use of diffusion tensors [20]. Existing nonlinear diffusion filters use a scalar diffusivity rather than a diffusion tensor [21, 22], and as such, they can be regarded as isotropic filters. CED allows for true anisotropic diffusion by building on the work of Cottet and Germain [23] and examining the eigenvalues and eigenvectors of the diffusion tensor. This means that the smoothing process locally adapts and allows smoothing in different directions. This makes CED well suited for myofibre segmentation as it allows for the intra-fibre regions (i.e. those regions contained “within” a myofibre) to remain coherent whilst enhancing the connective structures such as the perimysium and endomysium.

**Figure 2:**
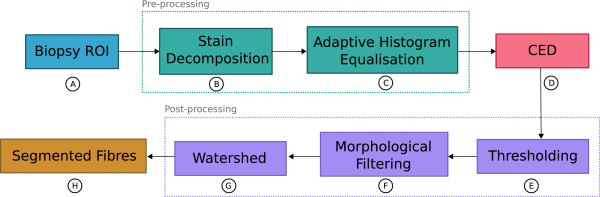
**Flow-diagram of the proposed myofibre segmentation methodology.** Each region of interest is initially pre-processed before Coherence-Enhancing Diffusion filtering is applied. Morphological post-processing is then used to clean the segmentation and remove any remaining noise. The result of performing each step **A–H** on a sample region of interest is shown in Figure 3 **A–H**.

**Figure 3:**
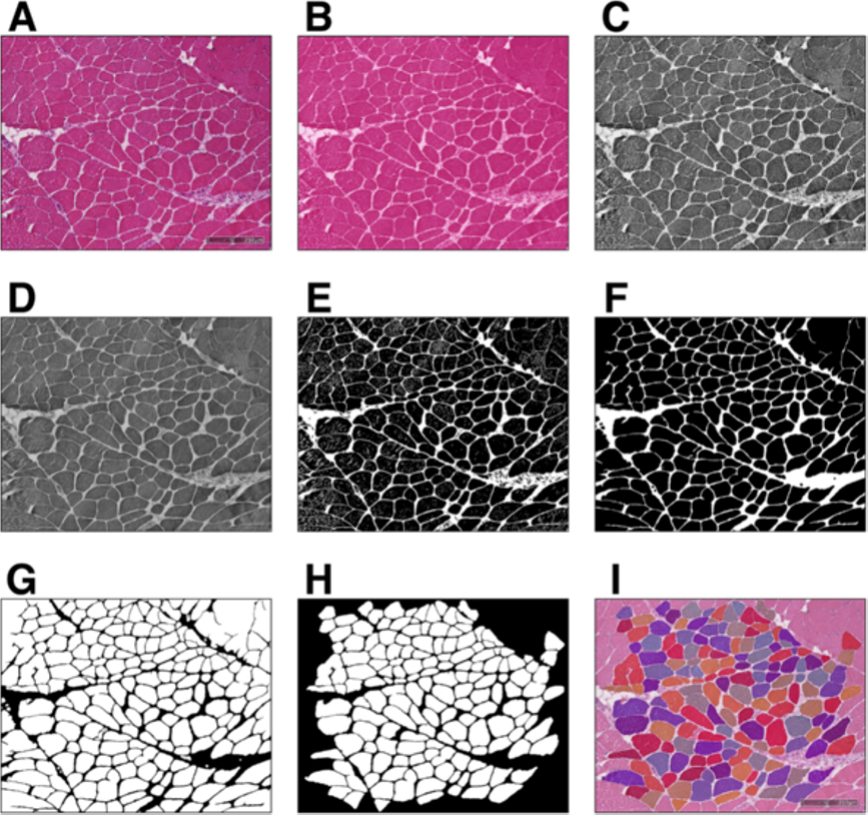
**Examples of the steps taken to perform myofibre segmentation using the proposed method.** The labels **A–H** refer to the steps outlined in the flow diagram in Figure 2 and panel **I** shows the final segmentation superimposed on the original image.

The proposed method can be coarsely divided into four main steps: 1) pre-processing, 2) Coherence-Enhacing Diffusion filtering, 3) morphological filtering, and 4) watershed transform. A flow-chart of this process is shown in Figure 2. The pre-processing step seeks to enhance the myofibre regions by performing stain decomposition and extracting only the eosinophilic structures from the image. As well as this, the myofibre boundaries are further enhanced by performing histogram equalisation on a grayscale version of the eosinophilic image. CED filtering is then applied on this pre-processed image. The purpose of the CED filtering step is to enhance the myofibre boundaries whilst also suppressing noise within the myofibre regions. Once CED filtering has been performed, a set of morphological operations are applied to the image. These operations seek to remove any small regions that correspond to noise and also to join together any disconnected boundary regions. The final step is to perform marker controlled watershed segmentation to split any touching myofibres and produce the final segmentation. Figure 3 shows an example image along with the CED image, the resultant myofibre boundaries, and the final segmentation.

### Pre-processing

The first step is to apply a stain decomposition method [24] to the muscle sections so that processing is performed on only the eosinophilic structures — in this case myofibres — thus excluding any nuclear regions from the segmentation process. This stain decomposition step ensures that the most prominent structures in the image are the myofibres themselves rather than any satellite nuclei. Numerous methods exist to perform stain decomposition, or colour un-mixing, however, the method used in this work does not require user input to select different regions corresponding to different staining. Instead, non-negative matrix factorisation is applied to find a factorisation of the original RGB image into two separate images, one corresponding to the eosinophilic image and the other to the haematoxylin image. As previously mentioned, the eosinophilic image is used and is converted to grayscale prior to having contrast enhancement performed using an adaptive histogram equalisation method [25]. The purpose of this step is to further enhance the myofibre boundaries prior to performing Coherence-Enhancing Diffusion filtering.

### Coherence-Enhancing Diffusion (CED) filters

The purpose of performing Coherence-Enhancing Diffusion filtering [14] is to enhance the myofibre boundaries in the pre-processed image. The pre-processed image, found using the methodology described above, is a grayscale image with lighter regions corresponding to the myofibre boundaries and darker regions corresponding to the myofibres. CED filtering seeks to enhance the lighter regions that correspond to boundaries whilst smoothing the interior regions that correspond to the myofibres.

The central step of CED is to determine the directions along which the image should be smoothed, this is achieved by utilising the eigendecomposition of the second moment matrix, or structure tensor. As explained in [20], the heart of anisotropic diffusion filtering using a diffusion tensor is the evolution of an image using
1

where *u*(*x*,*t*) is the image under evolution at diffusion time *t*. The diffusion tensor, *D*, is a positive semidefinite matrix which is adapted to the local image structure. The local image structure can be measured using the structure tensor [20, 26] which is defined as
2

where *u*_*σ*_ is defined as *u* convolved with a Gaussian with standard deviation *σ* such that *u*_*σ*_=*G*_*σ*_∗*u*. As such, *u*_*σ*_ is a regularised version of *u* obtained via convolution with a Gaussian *G*_*σ*_. The standard deviation, *σ*, can be thought to represent the *noise scale* since it forces the edge detector to ignore details smaller than *O* (*σ*) [14]. The standard deviation, *ρ*, defines the *integration scale* which describes the characteristic size of the texture [20]. The eigenvectors of *J*_*ρ*_ describe the local orientations, or coherence orientations, and the eigenvalues describe the average contrast along the eigendirections. The eigenvalues of  are


These eigenvalues are used to assemble the eigenvectors of the diffusion tensor *D*. The diffusion tensor of coherence-enhancing diffusion uses the same eigenvectors as *J*_*ρ*_ and the eigenvalues are assembled such that
34

where *c*_1_ ∈ (0,1) serves as a regularisation parameter that ensures that the diffusion tensor remains uniformly positive definite, and *c*_2_ > 0 serves as a threshold parameter.

The solution to Eq. 1 can be obtained numerically by utilising finite differences using a time step of size, *τ*, as the difference between two calculated time levels. For full details on the implementation of CED filtering the reader is pointed towards Section 5 of [14] and Section 2.2 and 3 of [20].

### Morphological filtering and watershed transform

The output of the Coherence-Enhancing Diffusion step described above is a grayscale image with enhanced myofibre boundaries and smoothed interiors. Although the fibre boundaries in general will be well connected, due to inherent noise within the images further processing needs to be performed to connect any disconnected boundaries and remove any remaining noise. The image produced from CED can be thought of as a probability map describing the likelihood of a pixel belonging to a boundary region. As such, to convert the given image into a binary segmentation a thresholding method is used to identify only the pixels corresponding to boundary regions. This threshold is automatically estimated using the well known Otsu method [6]. Following a similar methodology to [8], once the boundary segmentation has been produced, connected components with a total area of less than 750 pixels (674 *μm*) are removed. This ensures that any erroneous regions corresponding to staining or preparation artefacts are ignored from the final segmentation. The final morphological filtering step is to perform morphological closing [27] so as to join together any disconnected boundary regions. For the closing operation a disk structuring element of radius 4 pixels (3.6 *μm*) is used. The complement of this binary image then gives the segmentation of the myofibres. The final step is to apply a marker controlled watershed transformation [28] to split any touching fibres. Prior to applying the watershed transform the foreground markers are identified by eroding the binary image by 10 pixels (8.99 *μm*), the Euclidean distance transform of this binary image is then used to compute the watershed transform.

### Ethical approval

Use of human tissue is covered by a research tissue bank ethical approval which covers the diagnostic archive from which the material was drawn. The BioBank reference number is ACP000090. Use of material also conforms with Codes of Practice of the Human Tissue Authority.

## Evaluation

To assess the effectiveness of the proposed method it is tested against various existing myofibre segmentation algorithms using different measures of accuracy. Multiple evaluation criteria need to be employed as a single measure of accuracy is not appropriate and could lead to an unfair or biased view of an algorithm’s performance. As such, multiple criteria are used to build a bigger picture of the effectiveness of a segmentation algorithm. As well as this, the effect of each of the proposed method’s parameters are investigated and discussed.

### Evaluation criteria

For each method, the segmentation accuracy is measured using both image segmentation evaluation criteria and clinical morphometric measures.

#### Segmentation accuracy

The overall segmentation accuracy of a given method is measured as the number of correctly segmented myofibres (in relation to the given ground truth) divided by the total number of true positive, false positive and false negative regions. To ensure that a segmented myofibre is not counted across multiple regions, a one-to-one mapping is enforced between the ground truth and the automatic segmentations. Each ground truth region is mapped to a single segmented region that is encompassed within the ground truth boundary. Any remaining regions are then counted as false positives.

#### Fragmentation and congealment

To evaluate whether or not the different algorithms over or under segment the myofibre regions two new measures are proposed: *fragmentation* and *congealment*.

Fragmentation is used to measure to what extent an algorithm fragments a myofibre in the output segmentation. Given the ground truth segmentations and the output segmentation, fragmentation is defined as *F* = *p*/*n*, where *p* is the total number of fragmented fibres in the output segmentation and *n* is the total number of fibres in the ground truth. The fragmented fibres are found by counting the number of connected components within a masked region of the output segmentation with the mask being set as each fibre in the ground truth segmentation.

Congealment is measured in a similar fashion to fragmentation, however, the purpose of congealment is to measure the number of segmentations that span across multiple fibre boundaries. As such, congealment is defined as *C*=*q*/*n*, where *q* is the total number of congealed fibres in the output segmentation and *n* is the total number of fibres in the ground truth. A congealed fibre is found by counting the number of connected components within a masked region in the ground truth segmentation with the masks corresponding to the regions in the output segmentation.

For both fragmentation and congealment a value of 0 indicates a low fragmentation or congealment factor. As the value increases, the quality of the segmentation decreases since the algorithm is seen to fragment or congeal the segmentation.

#### Cumulative distribution functions

To estimate how well each algorithm performs with respect to the ground truth a measure is used based on the cumulative distribution function of misclassification errors [29]. For a given algorithm, the set of misclassification percentages, *S* = {*p*_*i*_,*i* = 1,…,*n*}, represents the set of all pixel based misclassifications on a per image basis such that, *p*_*i*_, represents the percentage of misclassified pixels for the *i*-th image with respect to the ground truth segmentation. A pixel is considered a misclassified pixel if it appears as a 1 in the ground truth but a 0 in the segmentation, or similarly, if it appears as a 0 in the ground truth and a 1 in the segmentation. Each algorithm will have a unique distribution of misclassification percentages and the distribution of misclassifications can be characterised by its cumulative distribution function (CDF). The CDFs are maximum likelihood estimators and are defined as
5

where *f*_*K*_ is the kernel density estimate of the histogram estimate based on *S*. The optimal algorithm has a CDF which corresponds to the unit step function [29]. Therefore, the closer an algorithm’s CDF is to the unit step function the better its correlation with the ground truth segmentation. The CDF is a useful tool for estimating a consistent and unbiased estimate of an algorithm’s performance.

#### Morphometric measures

The two morphometric measures used to evaluate the segmentation algorithms are mean fibre diameter and the variability coefficient [3]. The mean fibre diameter is measured as the length of the minor axis of the segmented fibre region. The minor axis is used as this is the measurement least affected by any kinking of the muscle as a result of processing or by any obliquity of the plane of section [2]. The variability coefficient is used to measure the amount of variability among the fibres and is defined as
6

where  and  are the standard deviation and mean respectively of the set of all fibre diameters for a given segmentation method. A variability coefficient of less than 250 is considered to be normal and any value above this is then considered abnormal [3]. Since the dataset is drawn from a normal population the variability coefficient should be less than 250.

### Parameter considerations

The initial parameters for the CED algorithm were set according to the values laid out and discussed in [20] and subsequently tuned to improve the results. The initial parameter estimates were, *τ* = 1,*σ* = 1 × 10^-4^ px (0.89 *μm*),*ρ* = 4 px (3.56 *μm*),*c*_1_ = 1 × 10^-10^,*c*_2_ = 1 × 10^-3^. The relative influence of each parameter was then assessed by varying the value of each parameter and assessing the change in the accuracy of the segmentation. A generalised linear regression model [30] was then fitted to the data to assess the relative influence of each parameter. The results are displayed in Table 1. As in [8], influence here refers to the relative effect the parameter has on the final segmentation quality. A low influence would suggest the parameter has little to no effect on the segmentation, where as a medium influence indicates that adjustments to this parameter can lead to noticeable changes to the segmentation in terms of accuracy. Only the integration scale, *ρ*, has a medium influence on the final segmentation quality. This is not surprising as *ρ* controls the characteristic size of the texture regions [20]. As such, selecting a sub-optimal value of *ρ* will lead to either a lack of coherence within myofibres such that the “noisy” aspects within the fibres are not smoothed, or the coherence-enhancement occurs over too large a scale so the fibre boundary details are destroyed. As can be seen in Table 1, all other parameters have a relatively low influence on the final segmentation quality. It is worth noting that when training on new data, it is expected that only the integration scale *ρ* will need tuning since the other parameters have a low influence on the final segmentation quality.

**Table 1:** **A description of the different parameters employed for Coherence-Enhancing Diffusion filtering along with their relative influence**

Parameter	Description	Influence
*τ*	Size of diffusion time step	Low
*σ*	Noise scale (Eq. 2)	Low
*ρ*	Integration scale (Eq. 2)	Medium
*c*_1_	Regularisation parameter (Eq. 3)	Low
*c*_2_	Eigenvalue threshold (Eq. 3)	Very low
*t*_area_	Minimum fibre area	Low

## Results

**Figure 4:**
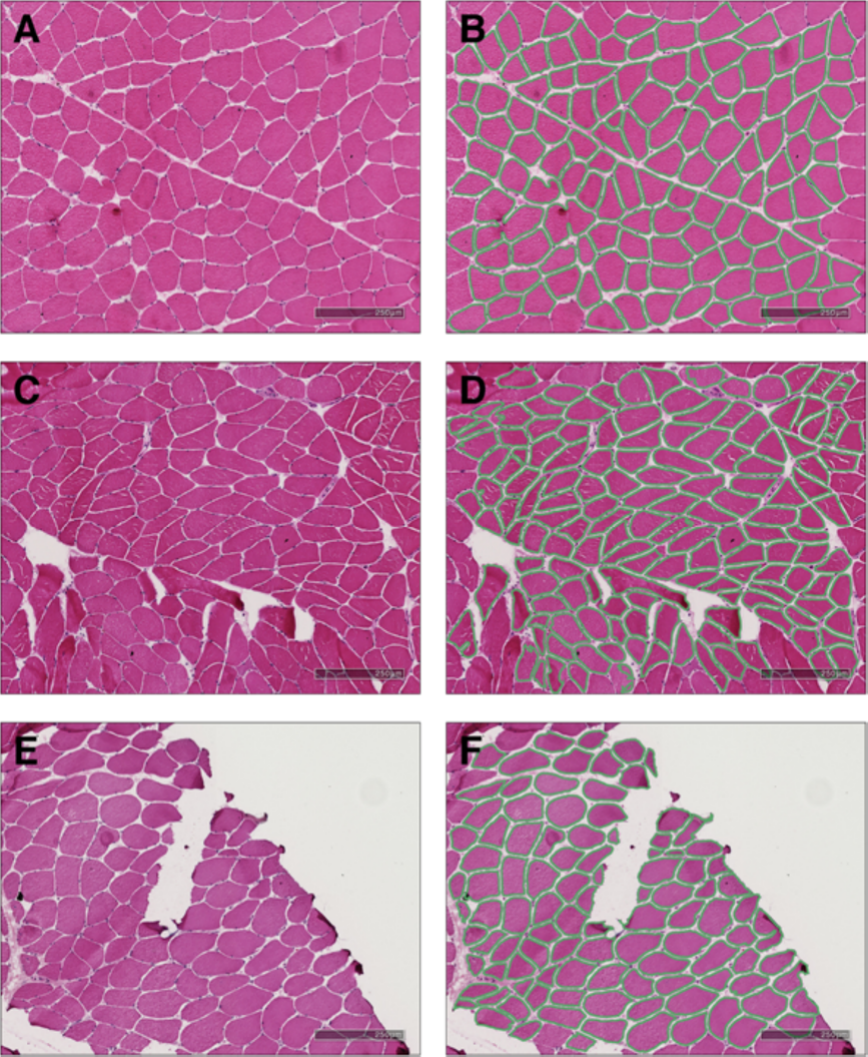
**Example ROIs (A, C, E) and segmentations (B, D, F) obtained using the proposed Coherence-Enhancing Diffusion approach described in this paper.**

Figure 4 shows example segmentations of three cases obtained using the proposed methodology. As can be seen, the myofibres are segmented with little to no inaccuracies in the segmentations. To quantify the accuracy of the proposed approach the above evaluation criteria are used and the performance is compared against existing methods.

### Comparative results

The proposed method is compared against basic thresholding [6], ensemble of line detectors [7], line enhancement using Frangi filters [9], hybrid level sets [31], and Chan-Vese active contours [32]. For the hybrid level sets approach, the algorithm was run for 200 iterations and the lower bound grey level was set to *μ* = 100. The propagation weight was set to 0.1 and the advection term was set to 500. For Chan-Vese active contours, the algorithm was run for 500 iterations and the weight of length term was set to *μ* =0.1. In both cases, an initial circular mask was used. The segmentations obtained from all methods are post-processed to remove any segmented fibres on the boundary of the image. As well as this, any segmented region smaller than 750 pixels in area (674 *μm*) is removed and a watershed segmentation is performed to split any touching fibres. This ensures that the segmentations are in keeping with the ground truth and also enables more accurate morphometric measurements to be taken. It also ensures that the comparisons are focused on the core algorithms as opposed to any post-processing method used. It is worth noting that the majority of works published on myofibre segmentation include these post-processing steps (e.g. [8, 12]).

**Figure 5:**
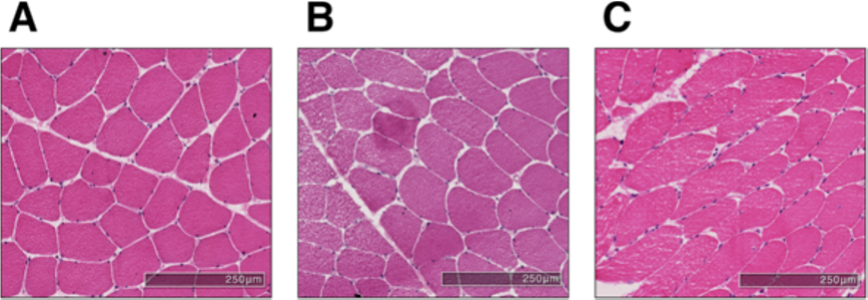
**Example cases drawn from the set of (A) low, (B) medium, and (C) high noise cases.** As shown in Table 3, most methods will perform well on the low noise cases, but the presence of noise within the myofibres combined with weak fibre boundaries **(B, C)** can lead to poor segmentation results.

The results of performing myofibre segmentation are displayed in Table 2 and show the efficacy of the proposed CED filtering method for myofibre segmentation. The results in Table 2 show that the CED method achieves a higher segmentation accuracy with a lower standard deviation. This suggests that the proposed method is more robust to the variation in appearance of fibres within the dataset. As well as this, the difference in performance of the proposed approach and the next best performing (hybrid level sets) is statistically significant with a *p*-value of *p* = 0.01. Many images within the dataset will contain fibres with weak boundaries and prominent myofibrils which is equivalent to “noisy” regions. When the myofibre regions are well defined with a strong endomysium or perimysium then all methods are able to segment the myofibres adequately. However, the data contains many examples where the myofibres are heterogeneous in appearance, due to problems with the staining (see Figure 5). Of the total 2814 fibres, 1222 were identified as ‘low’ noise, 882 were identified as ‘medium’ noise, and 710 were identified as ‘high’ noise. Table 3 shows the performance of the different algorithms under these three conditions. As can be seen, under low noise conditions all algorithms, except thresholding, perform well with accuracies of over 80*%* with little variation. However, under medium noise the performance of all algorithms drops and under high noise all algorithms, except the proposed CED method, drop to below 65*%*. CED is still able to accurately segment 81*%* of the fibres under high noise conditions. This is in contrast to the next best performer, hybrid level sets, which achieves an accuracy of 62*%* under high noise conditions. The reason for CED’s performance can be attributed to the algorithm’s ability to reduce noise *within* a myofibre whilst seeking to enhance the boundary lines. This means that it is less likely to fragment or congeal the fibres. Although the proposed CED method does not have the lowest fragmentation or congealment value, it does have the lowest combined fragmentation and congealment score of 0.18 (see Table 2). The next best performer is Chan-Vese active contours with a combined fragmentation and congealment value of 0.26. This shows that the proposed method is able to provide a robust segmentation with regions not being over or under segmented. This is in contrast to the worst performing method, thresholding, where some regions are badly fragmented or heavily congealed. A high value of both fragmentation and congealment indicates that the method is sensitive to heterogeneous areas, and so fragments these areas, and also to weak fibre boundaries leading to large congealed areas.

**Figure 6:**
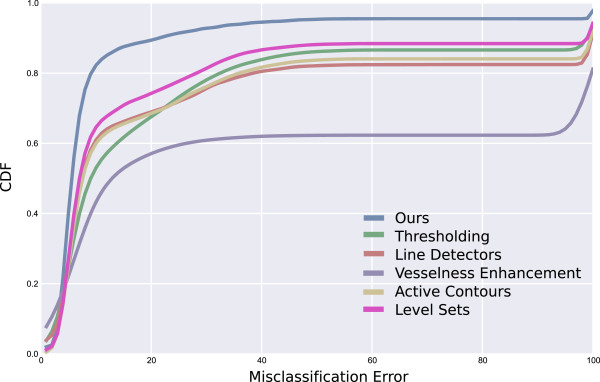
**Cumulative distribution functions (CDFs) of the different segmentation methods.** Each CDF corresponds to the per-pixel based classification performance of the given algorithm. The closer the CDF is to the unit step function the closer the segmentation is to the ground truth.

The cumulative distribution functions (CDFs) of the different methods are shown in Figure 6. The CDFs provide a consistent and unbiased estimate of how well the different algorithms perform on a per-pixel basis when compared to the ground truth segmentations. The closer the CDF is to the unit step function the closer the segmentation is to the ground truth. Figure 6 shows that the proposed approach outperforms other methods. The vesselness enhancement method performs poorly because although it identifies fibres these fibres do not closely match the ground truth segmentations.

**Table 2:** **Quantitative results for each segmentation algorithm**

	Fibre acc. ( *%*)	Fragmentation	Congealment	Mean diameter ( *μ* *m*)	VC ( *μ* *m*)
Thresholding [6]	66.97 (±20.75)	0.43 (±0.30)	0.35 (±0.28)	60.53 (±21.75)	337.10 (±92.60)
Line detectors [7]	68.16 (±19.61)	0.08 (±0.11)	0.19 (±0.13)	66.01 (±22.32)	336.00 (±139.22)
Vesselness enhancement [9]	63.90 (±23.42)	0.28 (±0.19)	0.07 (±0.12)	53.40 (±19.63)	312.84 (±58.20)
Hybrid level sets [31]	76.93 (±18.12)	0.09 (±0.09)	0.18 (±0.13)	63.41 (±21.48)	327.64 (±105.00)
Chan-Vese AC [32]	72.62 (±15.93)	0.06 (±0.08)	0.20 (±0.13)	66.41 (±22.21)	325.03 (±103.71)
Proposed method	88.65 (±8.58)	0.08 (±0.07)	0.10 (±0.11)	62.83 (±18.31)	251.85 (±59.74)
Ground truth				59.21 (±15.58)	230.31 (±40.07)

**Table 3:** **Fibre segmentation accuracies obtained after splitting the data into three groups according to the ‘noise’ level of each image**

	Low ( *%*)	Medium ( *%*)	High ( *%*)
Thresholding [6]	79.7 (±0.1)	77.2 (±0.2)	48.5 (±0.2)
Line detectors [7]	81.4 (±0.1)	79.2 (±0.2)	48.6 (±0.1)
Vesselness enhancement [9]	85.8 (±0.1)	65.4 (±0.2)	46.1 (±0.2)
Hybrid level sets [31]	87.3 (±0.1)	84.7 (±0.1)	62.4 (±0.2)
Chan-Vese AC [32]	84.0 (±0.1)	79.8 (±0.1)	57.8 (±0.1)
Proposed method	95.9 (±0.0)	91.1 (±0.0)	81.1 (±0.1)

When considering the clinical metrics, all methods achieve a mean diameter that is within the standard deviation range of the ground truth segmentations (see Table 2) so what is of more interest is the differences in the variability coefficient. As explained previously, the variability coefficient is a measure of the variation in fibre diameters across all segmented regions. As shown in Eq. 6, the variability coefficient assigns more prominence to the standard deviation of the measurements and so a method that has a large amount of variation will obtain a high variability coefficient score. It is noted that a variability coefficient score of below 250 is counted as normal [3], and since the data is drawn from a normal population all algorithms are expected to produce results with a variability coefficient of less than 250. This is validated by the ground truth which has a variability coefficient of 230.31 (±40.07). The proposed method has a variability coefficient of 251.85 (±59.74) which, although not below 250, is close enough to be counted as within range as it is less than 5*%* of the standard deviation out from the mark of 250. All other methods have a variability coefficient of greater than 300 which indicates abnormal cases, even though the data is normal. The reason for this large variability coefficient can be attributed to high fragmentation and congealment factors which lead to outliers within the segmentations as shown in Table 2.

**Figure 7:**
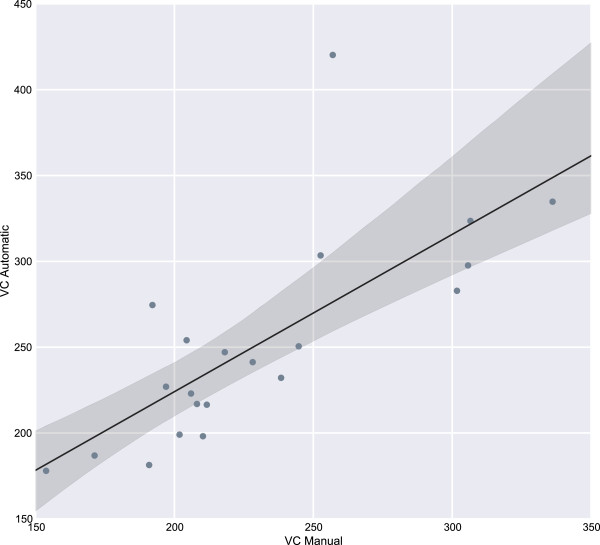
**Correlation of the variability coefficient between the ground truth segmentations and the proposed approach.** The correlation between the two is *r* = 0.74 indicating a very strong positive correlation.

Figure 7 shows the correlation between the variability coefficient of the ground truth segmentations and the segmentations found using the proposed approach. The Pearson’s R value of *r* = 0.74 indicates a very strong positive correlation between the ground truth variability coefficient and the variability coefficient found using the proposed approach. The outlier seen at the top of Figure 7 corresponds to a high-noise case. The CED method achieves a segmentation accuracy of 73*%* for this case which, although low, is significantly higher than the average for the image across the other methods (37.34*%*±14.57). As well as this, the mean variability coefficient for this case across the other methods is 553.82 (±119.31) compared to 420.23 for the proposed method.

### Failure cases

**Figure 8:**
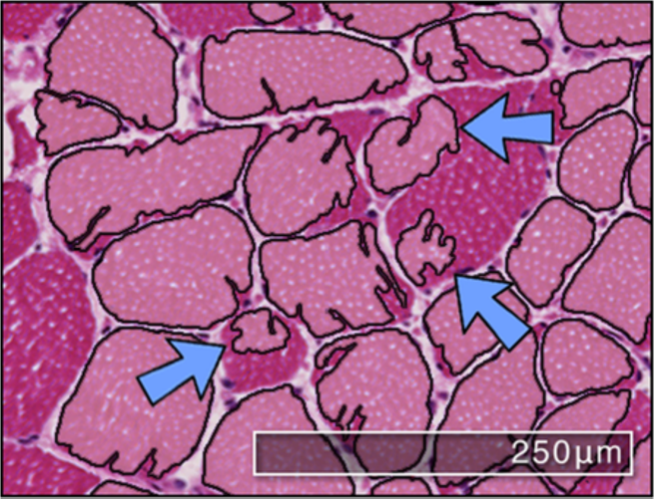
**Example failure case.** An example region of interest from a larger section where inadequate segmentations can occur for the proposed CED method. The blue arrows indicate myofibres that are poorly segmented and could lead to inaccurate morphometric measurements.

The segmentation in Figure 8 shows a cropped example case where the proposed CED method fails to adequately segment the myofibres. Two fibres in particular are badly segmented due to a weak boundary between the fibres. Although parts of each of the fibre have been identified, they are not useful for subsequent analysis and so count as a failure case. The reasons for such bad segmentation can be attributed to the fact that a single set of parameters are defined for the CED equations across all types of images. As mentioned previously, histological muscle biopsy images can be obstructed by the presence of noise due to incorrect staining or section handling, as such a single set of parameters may not be able to adequately account for all the variation in the data. It would be beneficial for future research to be focussed on providing an adaptive parameter methodology to handle such scenarios.

## Discussion and conclusions

A new method for myofibre segmentation in adult skeletal muscle cases has been presented based on Coherence-Enhacing Diffusion (CED) filtering. This proposed method allows for the myofibre boundaries to be enhanced whilst maintaing the coherence of the pixels within a myofibre region. The evaluation shows that when compared against existing methods for myofibre segmentation such as hybrid level sets, Chan-Vese active contours, and line enhancement, CED is able to achieve superior segmentation accuracies of approximately 89*%*. As well as this, through the use of morphometric features such as mean fibre diameter and the variability coefficient, the proposed CED method is able to closely match ground truth segmentations and produce results that are clinically consistent.

The implementations were done in MATLAB and run on an Apple iMac with a 3.4 GHz Intel Core i7 processor with 16 GB or RAM. The average run-times for each method is shown in Table 4. As can be seen, the proposed method is faster than the deformable model based approaches but slower than vesselness enhancement, edge detection, and thresholding. It is worth noting however that this is only an experimental implementation. Significant speedups could be made by implementing the proposed approach in C or C++. This would then provide a means for applying the segmentation method to whole-slide images and would pave the way for one area of future work.

**Table 4:** **The average run times for each of the proposed algorithms to segment an image of size 1300×1030 pixels (926×1169** ***μ*** ***m*** **)**

	Average run time (s)
Thresholding [6]	1.48 (±0.02)
Line detectors [7]	3.37 (±0.13)
Vesselness enhancement [9]	2.62 (±0.05)
Hybrid level sets [31]	76.60 (±3.40)
Chan-Vese AC [32]	123.31 (±1.73)
Proposed method	8.99 (±0.52)

Other future work will be focused on applying the proposed CED algorithm to abnormal cases so as to build a complete framework for myofibre segmentation and abnormality detection. Moreover, morphometric measurements will be used to automatically identify clinically interesting aspects such as atrophy and hypertrophy factors as well as fibre type proportions and prominence. However, to achieve this the proposed method will need to be tested and evaluated on ATPase stained sections. As well as this, future research will investigate employing CED filtering to whole slide images so as to obtain an estimate of fibre size variation across the full area of the biopsy. To achieve this, an automatic region detection algorithm will be employed to select the best possible regions for analysis. This will in turn reduce the number of segmentation errors since analysis will only be performed on “clean” regions.

In conclusion, an accurate technique has been presented for segmenting adult myofibres in H&E stained cases. The technique was evaluated on a number of representative regions and was compared against existing approaches. The results show that the proposed approach is able to show significantly improved results over current methods.
